# Development and validation of an immunoperoxidase antigen detection test for improved diagnosis of rabies in Indonesia

**DOI:** 10.1371/journal.pntd.0006079

**Published:** 2017-11-13

**Authors:** Ibnu Rahmadane, Andrea F. Certoma, Grantley R. Peck, Yul Fitria, Jean Payne, Axel Colling, Brian J. Shiell, Gary Beddome, Susanne Wilson, Meng Yu, Chris Morrissy, Wojtek P. Michalski, John Bingham, Ian A. Gardner, John D. Allen

**Affiliations:** 1 Balai Penyidikan dan Pengujian Veteriner Regional II Bukittinggi, Baso, Sumatera Barat, Indonesia; 2 CSIRO Australian Animal Health Laboratory, Geelong, Victoria, Australia; 3 Atlantic Veterinary College, Charlottetown, Prince Edward Island, Canada; Wistar Institute, UNITED STATES

## Abstract

Rabies continues to pose a significant threat to human and animal health in regions of Indonesia. Indonesia has an extensive network of veterinary diagnostic laboratories and the 8 National laboratories are equipped to undertake diagnostic testing for rabies using the commercially-procured direct fluorescent antibody test (FAT), which is considered the reference (gold standard) test. However, many of the Indonesian Provincial diagnostic laboratories do not have a fluorescence microscope required to undertake the FAT. Instead, certain Provincial laboratories continue to screen samples using a chemical stain-based test (Seller’s stain test, SST). This test has low diagnostic sensitivity, with negative SST-tested samples being forwarded to the nearest National laboratory resulting in significant delays for completion of testing and considerable additional costs. This study sought to develop a cost-effective and diagnostically-accurate immunoperoxidase antigen detection (RIAD) test for rabies that can be readily and quickly performed by the resource-constrained Provincial laboratories. This would reduce the burden on the National laboratories and allow more rapid diagnoses and implementation of post-exposure prophylaxis. The RIAD test was evaluated using brain smears fixed with acetone or formalin and its performance was validated by comparison with established rabies diagnostic tests used in Indonesia, including the SST and FAT. A proficiency testing panel was distributed between Provincial laboratories to assess the reproducibility of the test. The performance of the RIAD test was improved by using acetone fixation of brain smears rather than formalin fixation such that it was of equivalent accuracy to that of the World Organisation for Animal Health (OIE)-recommended FAT, with both tests returning median diagnostic sensitivity and specificity values of 0.989 and 0.993, respectively. The RIAD test and FAT had higher diagnostic sensitivity than the SST (median = 0.562). Proficiency testing using a panel of 6 coded samples distributed to 16 laboratories showed that the RIAD test had good reproducibility with an overall agreement of 97%. This study describes the successful development, characterisation and use of a novel RIAD test and its fitness for purpose as a screening test for use in provincial Indonesian veterinary laboratories.

## Author summary

In Indonesia, veterinary diagnostic laboratories conduct tests for rabies on brain samples from animals suspected of being infected with rabies virus. National laboratories use internationally recommended tests for rabies virus that require expensive materials and equipment. Remote and smaller Provincial laboratories use a simpler older-generation chemical stain test that is less costly to perform but is also highly inaccurate resulting in many rabies-infected brains returning false negative test results. Brain samples that give negative results at a Provincial laboratory are then transported to a National laboratory for retesting to confirm the diagnosis. This results in additional costs and time delays and creates the need for a more effective, lower cost rabies test that Provincial laboratories can effectively use. This paper describes the development of one such test that is of comparable accuracy to the internationally recommended test for detecting rabies in brain and does not require expensive equipment to perform.

## Introduction

Rabies is a lethal zoonotic viral disease caused by a member of the *Lyssavirus* genus within the *Rhabdoviridae* family. Dog bites are responsible for transmission of rabies to humans in 99% of all mortalities and for 90% of post-exposure prophylaxis (PEP) globally. Rabies was first reported in Indonesia in 1884 and is thought to be endemic in 24 of the country’s 34 provinces [1] where it causes 150 to 300 human fatalities annually [2]. Indonesian rabies isolates belong to Asian lineage within the classical rabies virus, *lyssavirus* genotype 1 [3]. Control programs at provincial and district levels are regularly implemented in Indonesia but adequate vaccination coverage has been difficult to achieve.

**Fig 1:**
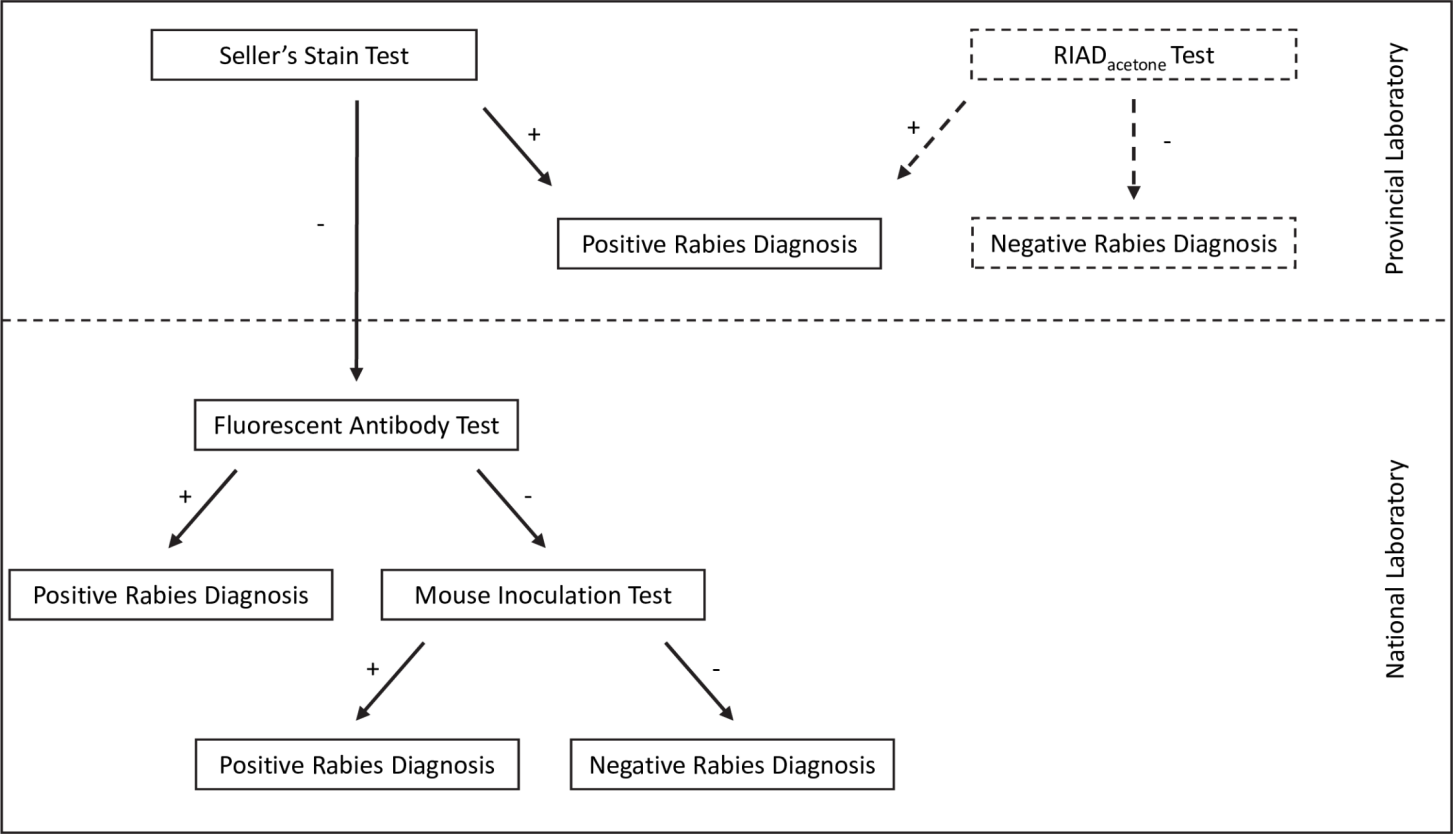
Flowchart showing current and proposed (dashed lines) laboratory testing regimen for diagnosis of rabies in Indonesia following competency assessment of individual provincial laboratories using the RIAD_acetone_ test.

Testing of suspected animal rabies cases is conducted at Indonesian veterinary service laboratories; 8 National and 23 Provincial. The direct fluorescent antibody test (FAT) is widely used as the reference test for rabies diagnosis due to its diagnostic performance [4]. The FAT requires expensive fluorescein isothiocyanate (FITC)-labeled antibodies and a well-maintained fluorescence microscope. As a result, Indonesia’s well-resourced National laboratories have the capacity to perform the FAT whilst the majority of Provincial laboratories must instead use the less accurate Seller’s stain test (SST) for preliminary diagnosis. SST involves chemical staining and microscopic observation for the presence of intra-cytoplasmic intra-neuronal inclusion bodies (Negri bodies) to indicate rabies infection [5]; however, SST has low diagnostic sensitivity [6, 7]. Brain samples that return a negative result using SST are sent to the nearest National laboratory for confirmatory follow-up testing by FAT and by mouse inoculation test (MIT) if the FAT returns a negative result (Fig 1). This results in considerable delays in reporting results and the additional testing of SST-negative samples (many of which are false negatives) places an unnecessary burden on the receiving National laboratory. Delays in confirming and reporting on rabies cases can lead to suboptimal bite case management.

To improve access to reliable rabies diagnostics, other rabies virus detection tests have been developed for use in settings where laboratory resources are limited. For example, a direct rapid immunohistochemical test (dRIT) that uses a cocktail of two biotin-labelled monoclonal antibodies (mAbs) to detect rabies antigen in brain has been described [8]. A modification of this dRIT that replaced the biotin-labelled mAbs with a biotin-labelled polyclonal antibody (pAb) demonstrated improved performance [8, 9]. Both tests eliminated the need for expensive fluorescence microscopes; however, the cost of producing primary antibody conjugates and the absence of their commercial supply could prevent widespread use of these tests. In some countries, commercially-produced rapid (lateral flow) tests are used in remote veterinary clinics for testing of brain samples from suspect rabid dogs. Test performance can vary depending on the specific commercial supplier [10] and, for countries such as Indonesia where provincial authorities have established veterinary laboratory networks, the use of such field-based tests is contrary to their desired objectives to build sustainable and cost effective capacity in laboratories. This paper describes the development and validation of an alternative pAb-based, indirect, non-fluorescent test that overcomes these supply constraints by replacing the use of biotinylated primary antibodies with an anti-rabies pAb used in combination with a commercially available horseradish peroxidase (HRP)-conjugated secondary antibody. In addition, the RIAD test uses light as opposed to fluorescence microscopy and has been produced and tested as a kit making it suitable for use in resource-limited laboratories.

## Methods

### Plasmids and cloning

Rabies challenge virus standard (CVS) strain nucleoprotein gene was subcloned into pETHb, a derivative of pET50b (Novagen) [11]. In brief, rabies virus nucleoprotein (RABV NP) gene was amplified by PCR using primers 5’-gaatggatcctacaatggatgccgacaaga and 5’- attcaagcttatgagtcactcgaatatgt and purified by elution from 0.8% (w/v) agarose gel. The PCR fragment and pETHb were digested with *Bam*HI and *Hind*III (Promega) and pETHb was dephosphorylated with TSAP (Promega). Both insert and vector were gel-purified and ligated with T4 DNA ligase (Promega). Resulting pET-RABV NP was used to transform DH5 alpha *Escherichia coli* (Invitrogen). The pET-RABV NP construct was purified from positive clones and sequence fidelity was confirmed by Sanger sequencing.

### Ethics statement

The Australian Animal Health Laboratory (AAHL) Animal Ethics Committee (AEC) is licensed with Agriculture Victoria, Australia, and complies with all the relevant requirements of the Prevention of Cruelty to Animals Act (186) and the Regulations; and complies with the Australian Code for the care and use of animals for scientific purposes (8th Edition 2013). The AAHL AEC approved the use of animals for production of antisera within the Small Animal Facility (SAF) at AAHL under AEC protocol number 1401.

### Sodium dodecyl sulfate (SDS) polyacrylamide gel electrophoresis (PAGE), Coomassie blue staining and immunoblotting

Protein samples were solubilized in NuPAGE 1× LDS sample buffer (Invitrogen) containing 50 mM dithiothreitol (DTT, Promega) (reducing sample buffer) by heating at 100°C for 10 min. Samples were loaded onto NuPAGE 4–12% Bis-Tris polyacrylamide gels and run in MOPs buffer (Invitrogen) at 200 V for 50 min. Gels were stained for 10 min in 0.25% (w/v) Coomassie Brilliant blue R250 then destained by washing for 20 min several times in a solution of 5% (v/v) acetic acid and 15% (v/v) methanol.

For immunoblotting, proteins from unstained gels were Western transferred to PVDF membrane (Pall) at 200 mA for 1 h in 20 mM N-cyclohexyl-3-aminopropanesulfonic acid (CAPS) buffer pH 11 with 10% (v/v) methanol then blocked for 1 h in 30 mL 5% (w/v) skim milk in TBST (20 mM Tris pH 7.4, 150 mM NaCl, 0.05% (v/v) Tween 20). Primary rabbit antisera (produced in the SAF at AAHL as described below) and secondary HRP-conjugated goat anti-rabbit antibody (Bio-Rad) were diluted 1:10,000 and 1:20,000 in TBST, respectively, and applied separately to transfer membranes for 1 h each at room temperature (RT) on a rocking platform. Membranes were washed three times for 10 min per wash with 50 mL TBST after incubation with primary and secondary antibodies. Enhanced chemiluminescence using ECL Plus (Pierce) was used to detect immunoreactive bands as read using x-ray film or a Typhoon FLA9000 fluorescence scanner (GE).

### Rabies virus nucleoprotein expression

Chemically competent *E*. *coli* (Shuffle, NEB) were transformed with 10 ng purified pET-RABV NP as per manufacturer’s instructions. An individual colony from a RABV NP-expressing clone grown on selection media was used to inoculate a 10 mL LB broth starter culture containing 100 μg/mL ampicillin (Sigma). The culture was grown for 18 h at 30°C with agitation at 250 rpm and used to inoculate 1 L of LB broth containing 100 μg/mL ampicillin and grown under these conditions for approximately 3 h. When the culture reached an optical density of 0.6 at 600 nm, isopropyl-beta-D-thiogalactopyranoside was added to a final concentration of 0.4 mM to induce expression of the nucleoprotein and the culture was grown for a further 3 h or overnight at 16°C. Cells were separated from medium by centrifugation at 4,000 x *g* for 10 min at 4°C and cell pellets were stored at -80°C until required for further processing.

Cell pellets were thawed on ice then lysed on a rocking platform mixer for 20 min at RT in 50 mL BugBuster Master Mix (Novagen) containing protease inhibitors (P8849, Sigma). RABV NP inclusion bodies were purified from the lysate in diluted BugBuster Master Mix according to the manufacturer’s instructions. Inclusion bodies were resuspended and solubilized by heating to 100°C for 10 min in 750 μL NuPAGE 1× LDS sample reducing buffer.

### Rabies virus nucleoprotein purification

RABV NP concentration was approximated by comparison with bovine serum albumin (Sigma) standards resolved by SDS PAGE and stained with Coomassie Brilliant blue R250. Approximately 100 μg of solubilized RABV NP inclusion bodies was resolved by SDS PAGE on NuPAGE 4–12% Bis-Tris 1 mm x 2D preparative well gels (Invitrogen) at 200 V for 50 min. Gels were washed 3 times for 1 min with deionized water then overlaid with ice-cold 0.3 M KCl to visualize RABV NP protein bands. Bands were excised using a flexible skin graft knife blade then macerated by extrusion through a Luer lock syringe. RABV NP was isolated by adding one gel volume of phosphate buffered saline (PBS), 0.1% (w/v) SDS to the gel fragments and passively eluting overnight at RT on a rotating wheel mixer. The gel-buffer slurry was transferred to Microsep 0.2 μm Supor membrane centrifugal devices (Pall) and centrifuged at 4,000 x *g* for 5 min at RT. Flow-through containing eluted RABV NP was collected and the passive elution process was repeated once for 1 h to isolate any residual RABV NP. Isolated RABV NP fractions were pooled and protein concentration was determined using a BCA protein assay kit (Pierce). RABV NP was concentrated by centrifugation in a 3 kDa MWCO Centrifugal Filter Unit (Millipore). Purification of RABV NP was confirmed by SDS PAGE followed by staining with Coomassie blue and by immunodetection with penta-His monoclonal antibody (Qiagen) as per manufacturer’s instructions.

### Immunogen preparation and antisera production

Immunogen was prepared as a water-in-oil emulsion of purified RABV NP and CSIRO Triple Adjuvant prepared as described previously [12]. For each rabbit immunized, 600 μL of immunogen was prepared by mixing 90 μL of RABV NP at 1 mg/mL with 54 μL PBS and 96 μl of 3 mg/mL QuilA (Superfos Biosector), 30 mg/mL DEAE-Dextran (Pharmacia). This aqueous phase was added to 360 μL of Montanide ISA 50 V2 (Seppic) and emulsified by repeated extrusion through an 18 gauge blunt needle.

Two New Zealand White rabbits were immunized by intramuscular injection on three occasions approximately three weeks apart. Each immunization used a total of 75 μg RABV NP in two 0.25 mL doses, one dose in each hind leg. Serum samples (~1 mL) were taken prior to immunization to test for background staining. Sera were taken after each immunization and assessed for the presence of RABV NP antibodies by immunoblotting.

### Laboratory diagnosis of rabies

All brain samples used in this study were existing samples submitted to a National laboratory for testing as part of the Indonesian Government’s control measures for rabies initiative. Brain samples transported in 50% glycerine-saline solution were washed in PBS pH 7.4 for 30 min at RT, and tested using SST or the FAT. SST is a rapid method of staining brain tissue smears or sections that incorporates methylene blue and basic fuchsin dyes [13]. The FAT is a direct immunostaining method of acetone-fixed brain smears that utilizes FITC-conjugated anti-rabies antibody to detect viral antigen [14]. Commercially available anti-rabies FITC-conjugated antibody (Bio-Rad) was used for the FAT as per manufacturer’s instructions.

### RIAD method

Smears of brain material were prepared on positively charged (DAKO) or (3-aminopropyl) triethoxysilane (AAS; Sigma)-coated glass microscope slides. Smears were air dried for 5 min and fixed in 100% acetone (RIAD_acetone_) at -20°C for 30 min or neutral buffered formalin (RIAD_formalin_) for 30 min at RT. Smears were air dried again then treated with 200 μL of 3% (v/v) hydrogen peroxide for 10 min at RT in a humidified chamber. The rabbit primary anti-RABV NP antibody is described above. The secondary antibody was a commercial HRP-labelled anti-rabbit antibody (Envision Dako). All antibody incubations were performed at RT for 45 min in a humidified chamber. Brain smears were incubated sequentially with 200 μL of primary anti-RABV NP antiserum diluted 1:1000 and secondary HRP-labelled anti-rabbit antibody diluted 1:500. Dilutions were prepared using TBST containing 1% (w/v) skim milk (Australian origin). Smears were washed three times for two min per wash with TBST after each of the fixing, blocking and incubation steps. Chromogen was prepared immediately prior to use by adding 5 μL 30% (v/v) hydrogen peroxide to 500 μL of 4 mg/mL 3-amino-9-ethylcarbazole (Sigma) in N,N,dimethylformamide (Sigma) and diluting to 10 mL with 50 mM sodium acetate, pH 5. Brain smears were incubated in 200 μL of chromogen substrate for 10 min at RT and the reaction was stopped by rinsing once with distilled water. Smears were counterstained with Mayer’s haematoxylin (Lillie’s modification; Australian Bio Stains) for 20 s, rinsed once with distilled water followed by TBST then mounted with aqueous mounting medium (DAKO). Smears were viewed using transmitted white light microscopy with x20 or x40 objectives.

### Interpretation of results

Brain samples were deemed positive for rabies virus antigen if neuronal cytoplasmic green fluorescence was present in the FAT; neuronal cytoplasmic brick red deposits were seen for RIAD stained samples; or intracytoplasmic inclusion (Negri) bodies were detected using SST. In the absence of these indicators the sample was classified as negative.

### Validation samples

Dog brain samples (n = 116) were tested for exclusion of rabies virus at DIC Bukittinggi. The RIAD tests were assessed using this panel of samples derived from animals involved in human dog bites cases from within Sumatra and adjacent smaller islands that were submitted for diagnosis (“diagnostic group”) and another panel of 110 canine brain samples obtained from diagnostic samples submitted to the laboratory from Indonesian Government-administered dog population control activities in areas including Riau Island and districts within Bali that were thought to be free of rabies but which were close to endemic rabies areas (“survey group”). Both the diagnostic group and the survey group of samples were collected over a 2 year period up to 2015. Each sample was determined to be positive or negative using the SST, FAT or RIAD when assessed blind and in parallel by independent laboratory staff within the Disease Investigation Center (DIC) Bukittinggi.

### Statistical analysis

Diagnostic sensitivity (DSe) and specificity (DSp) were estimated for RIAD, FAT and SST using a 3 tests-in-2 population Bayesian latent class model (LCM) which allowed for conditional dependence in the sensitivities of RIAD and FAT [15]. Existing Bayesian code (http://cadms.ucdavis.edu/diagnostictests/2dep1ind3t2p.html) was modified for the analysis. The populations were the diagnostic and survey groups, where the latter was confined to dogs which had complete results on all 3 assays (n = 80). A sensitivity dependence (covariance) term was incorporated into the model to account for the fact that the RIAD and FAT assays target the same conserved protein of the rabies virus but probably different epitopes. The SST results were assumed to be conditionally independent of RIAD and FAT results because the SST uses a chemical stain to identify the presence of Negri bodies whereas the RIAD and FAT use rabies virus-specific antibodies to identify viral protein; hence, additional covariance terms were considered unnecessary. Two separate models were created for the RIAD test when used on slides fixed with acetone or formalin. A specificity covariance was not considered since all 3 tests gave zero positive results in the survey (presumed non-infected) population. Flat priors (beta 1,1) were used for DSe and DSp of all 3 tests and prevalence in the diagnostic group. For the survey population, one of the authors (JA) believed that there was a 10% chance that the population might be infected but he was 90% sure that if dogs were infected, prevalence would be <1% with a most likely value (mode) of 0.1%. The latter information equated to a beta (1.27, 274.82) prior. The opinion of JA was supported by two coauthors (IR, YF) who have extensive experience with rabies in Indonesia. Hence, prevalence in the survey group was modelled as a mixture distribution with a prevalence = 0% (point mass of 0) with 90% probability and a beta (1.27, 274.82) distribution with 10% probability.

Models were run in OpenBUGS 3.2.3 rev. 1012 [16, 17], and results were reported as medians and 95% probability intervals (PI). The difference in DSe of the 3 combinations of test pairs was calculated at each iteration of the model and the step function in OpenBUGS was used to estimate whether the difference in DSe (e.g. DSe _RIAD_ minus DSe _Sellers_) was positive. Briefly, the step function creates a Boolean variable (1 if positive, 0 if negative or zero) for any node (e.g. DSe) and the proportion of ones across all iterations can be interpreted as the probability (P) that a test has a higher DSe than a comparator test where P = 1 indicates certainty and P = 0.5 indicates no difference. The models were initially run for 50,000 iterations after the initial 5,000 iterates were discarded as burn-in. Model convergence was assessed by examination of history plots and running 2 separate chains from different initial values and plots of model parameters were checked for autocorrelation and thinning was done, if necessary. A sensitivity analysis was done to assess effects of a flat prior (beta 1,1) for prevalence in the survey group rather than use of the mixture distribution described in the previous paragraph.

### RIAD kit preparation

To determine the reproducibility of the RIAD test, RIAD test kits containing materials sufficient for 50 tests were delivered to 16 Provincial laboratories for their use against a proficiency test panel of dog brain samples. Kits included rabbit anti-RABV NP polyclonal antiserum pre-diluted in Envision FLEX diluent (DAKO), wash buffer (TBST), antibody diluent, anti-rabbit HRPO-conjugated secondary antibody (Jackson), AAS-coated slides, plastic ware, and positive and negative acetone-fixed dog brain control smears. Tests were performed according to protocols described above.

### Proficiency testing panel

Sixteen Indonesian Provincial veterinary laboratories used the RIAD test kit to assess a panel of unknown positive and negative samples (samples 1–6) derived from the hippocampus of 6 individual dogs. Four canine brain samples were rabies virus-positive (samples 1, 2, 4 and 5) and 2 were rabies virus-negative (samples 3 and 6). The proficiency testing (PT) organizing laboratory at DIC Bukittinggi provided all brain samples and determined their disease status using the FAT and RIAD test. Brain smears were prepared from frozen tissue that was thawed and passed 5 to 10 times through an 18 gauge needle. Homogenized brain was fixed in acetone, air-dried and treated with hydrogen peroxide, as described above, then stored at -20°C. Homogeneity of smears was tested at DIC Bukittinggi using the RIAD test prior and subsequent to the PT round. Stability of dog brain smears fixed in acetone had been previously ascertained during the development of the RIAD test and determined to be at least 6 months duration when stored at -20°C. The panel was transported chilled with the above mentioned kit.

## Results

### Expression and purification of RABV NP

**Fig 2:**
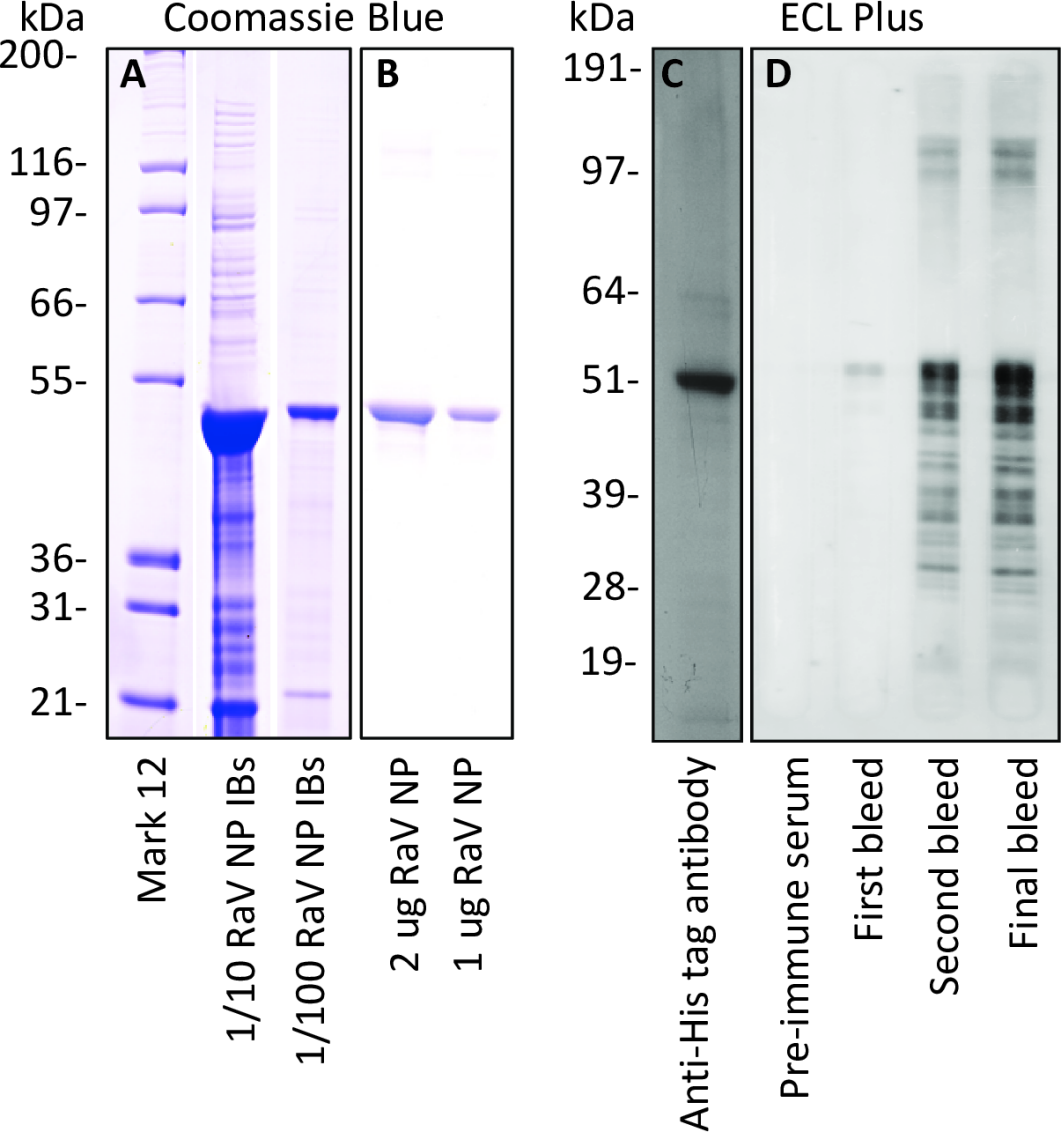
Expression and purification of RABV NP and characterization of antiserum. RABV NP inclusion bodies (IBs) (A) and gel-purified RABV NP (B) were resolved by SDS PAGE and stained with Coomassie blue. All lanes of gels stained with Coomassie blue were loaded with 10 μl of RABV NP in the dilutions or amounts indicated. Recombinant, gel-eluted His-tagged RABV NP was identified by immunoblotting with anti-His antibody (1:1,000) followed by sheep anti-mouse-HRP (1:2,000) (C). Sera from a pre- and post-immunized rabbit were diluted 1:10,000 and assessed for anti-RABV NP polyclonal antibody production by immunoblotting (D). All gels used for immunoblotting were loaded with 10 ng of RABV NP per well. Molecular mass markers were Mark 12 or See Blue Plus 2 (Invitrogen).

RABV NP was abundantly expressed in Shuffle *E*. *coli* and was partially purified from bacterial lysates as insoluble inclusion bodies (Fig 2A). Passive gel elution of the ~50 kDa RABV NP protein in the inclusion body preparation from polyacrylamide gels yielded an enriched RABV NP fraction as demonstrated by staining with Coomassie blue (Fig 2B) and by immunoblotting with penta-His antibody (Fig 2C).

### Generation of polyclonal RABV NP antiserum

Pre-immune rabbit sera showed no background reactivity to purified RABV NP by immunoblotting (Fig 2D). Serum samples taken approximately 14 days after each immunization showed successful anti-RABV NP seroconversion by immunoblotting against purified RABV NP and successive immunization led to greater anti-RABV NP polyclonal antibody titer (Fig 2D).

### Demonstration of rabies virus antigen in brain smears

**Fig 3:**
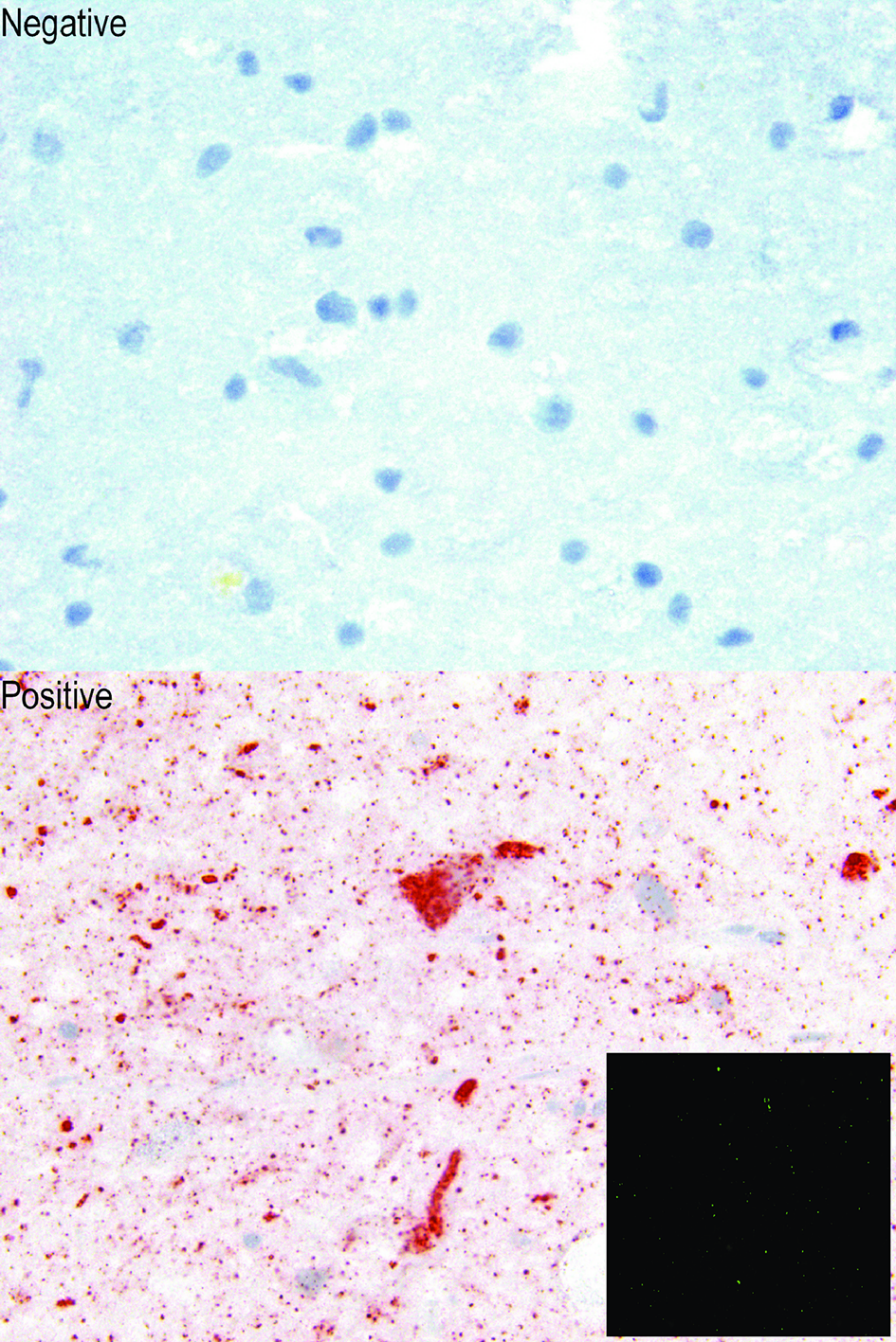
Comparison of RIAD_acetone_ test with FAT (inset) on canine brain smears infected with rabies virus (positive) or not (negative). The presence of RABV antigen is indicated by brick red deposits (Positive) or green fluorescence (inset) in brain smears tested using the RIAD_acetone_ test or FAT, respectively. Magnification of the RIAD_acetone_ images are 63X and the FAT image is 20X. All smears were fixed in acetone.

Rabies virus antigen detected with the RIAD in brain smears appeared as fine to globular red-brown particles within brain smear material (Fig 3). While much of the antigen was found free within smear material, it was also found within the cytoplasm of neuron cell bodies. The staining pattern and distribution of antigen in RIAD was similar to that found in the FAT. No red-brown particulate staining was seen in negative smears.

### Estimates of diagnostic sensitivity (DSe) and diagnostic specificity (DSp)

History and trace plots and based on use of 2 Markov chains indicated that all models converged. However, autocorrelation was evident in 3 plot: DSe for RIAD_formalin_ and FAT, and the sensitivity covariance between RIAD_formalin_ and FAT. For inferences about parameters, we ran 500,000 iterations thinned by 10 to minimize any effects of autocorrelation on estimates of these 3 parameters.

The RIAD_acetone_ test and FAT results were highly accurate, with both producing identical median DSe and DSp values of 0.989 and 0.993, respectively (Table 1). This was consistent with the empirical finding that there were no discordant test results. The resultant step function also indicated no difference in DSe (RIAD_acetone_ > FAT: P = 0.500). In the diagnostic group, the SST returned 44 negative results when both RIAD_acetone_ and FAT were positive indicating lack of sensitivity (median = 0.562). The step function showed that the RIAD_acetone_ and the FAT had greater DSe than the SST with probability of 100%. Comparisons of RIAD_formalin_, FAT and SST yielded similar findings (Table 1) except that RIAD_formalin_ was more sensitive than FAT in the step function analysis (P = 0.867) and reflected in a median sensitivity that was 3% higher. As expected, the estimated median prevalence of rabies in the human bite case (diagnostic) group in the 2 analyses was similar (0.869 and 0.908 for RIAD_acetone_ and RIAD_formalin_, respectively). Median estimates of the sensitivity covariances were close to zero (Table 1) indicating that they were of little practical importance. However, because the 95% probability interval for the RIAD_acetone_ model excluded zero and the 2.5 percentile for RIAD_formalin_ model was close to zero, the sensitivity covariance term was left in both models.

**Table 1:** Posterior medians and 95% probability intervals (PI) for diagnostic sensitivity (DSe) and specificity (DSp) from a 3-tests-in-2-population Bayesian latent class model.

		RIAD acetone-fixed smears (n = 116)		RIAD formalin-fixed smears (n = 115)	
Parameter		Median	95% PI	Median	95% PI
RIAD	DSe	0.989	0.950–0.9996	0.972	0.919–0.995
	DSp	0.993	0.962–0.9997	0.990	0.944–0.9996
FAT	DSe	0.989	0.950–0.9996	0.941	0.881–0.981
	DSp	0.993	0.962–0.9997	0.991	0.954–0.9997
SST	DSe	0.562	0.465–0.656	0.542	0.446–0.637
	DSp	0.993	0.962–0.9997	0.993	0.961–0.9997
Prevalence (human bite cases)		0.869	0.800–0.923	0.906	0.842–0.956
Prevalence (survey dogs)		0.002	0.0–0.011	0.002	0.0–0.012
Sensitivity covariance		0.003	0.00002–0.032	0.006	-0.001–0.039

All tests correctly identified each of 80 canine brain smears obtained from the presumed rabies-free regions as negative for rabies infection; hence step function results for differences in specificities between tests were not evaluated. Median DSp estimates for all tests were between 0.990 and 0.993. Use of a flat prior on prevalence (beta 1,1) in the survey group rather than an informative mixture prior had minimal effects on test performance characteristics but the median prevalence in the survey group was 4.5 times higher (median = 0.009, 95% PI = 0.0003–0.044) with use of the flat prior, and probability intervals for DSe and DSp of the 3 tests were slighter wider.

### Proficiency testing using RIAD test

The PT round showed that most laboratories were proficient in using the new RIAD_acetone_ test demonstrating its high reproducibility between participating laboratories (Table 2).

**Table 2:** Proficiency testing (PT) data using the RIAD_acetone_ test on 6 samples (4 positive and 2 negative for rabies infection) with 16 participating laboratories (A—P).

Sample	Participating laboratory																Assigned Result*	Agreement
	A	B	C	D	E	F	G	H	I	J	K	L	M	N	O	P		
1	+	+	+	+	+	+	+	+	+	+	+	+	+	+	+	+	+	16/16
2	+	+	+	+	+	+	+	+	**-**	+	+	+	+	+	+	+	+	15/16
3	-	-	-	-	**+**	-	-	-	-	-	-	-	-	-	-	-	-	15/16
4	+	+	+	+	+	+	+	+	+	+	+	+	+	+	+	+	+	16/16
5	+	+	+	+	+	+	+	+	+	+	+	+	+	+	+	+	+	16/16
6	-	-	-	-	-	-	-	**+**	-	-	-	-	-	-	-	-	-	15/16

* Assigned result obtained from multiple tests of samples 1–6 by the PT organising laboratory.

## Discussion

Rabies is a lethal zoonotic disease that is endemic in 24 provinces situated across numerous islands within the Indonesian archipelago [1]. The disease presents a significant economic burden to the region due to costs associated with diagnosis, treatment and control programs. Rabies infections in humans are often fatal and thus accurate and timely diagnosis is critical when human exposure is suspected, although the risk to humans is mitigated in Indonesia where the policy is to treat all potentially exposed humans. Nevertheless, the seriousness of the disease demands a thorough diagnostic testing regimen that ensures a low probability of obtaining false-negative results. To achieve this the current diagnostic strategy within Indonesia is to confirm negative results obtained using the SST with the FAT and any negative FAT results with the mouse inoculation test (MIT) such that a true negative is only confirmed after three tests are performed. The SST was demonstrated to have significantly lower diagnostic sensitivity leading to a high number of false-negative results (Table 1), but remains a frontline diagnostic test in resource-constrained laboratories due to its simplicity and affordability. This result was expected as the SST stains brain smears to detect the presence of Negri bodies in the cytoplasm of virus infected neurons [4]. This requires skill and patience in searching for the possible presence of Negri bodies compared with immunological detection of widely-dispersed viral antigen in brain smears [6]. For this reason the SST is not officially accepted by the OIE or World Health Organization as a diagnostic test for rabies providing further impetus for its replacement as the routine first line of testing in Provincial laboratories. The SST is inexpensive to perform, however, this possible economic benefit dissipates when a negative result is produced, be it true or false, since all negative samples must then be transported to National laboratories where they are retested using the more expensive FAT and possibly MIT. Therefore a low cost, simple to use, frontline test that has similar diagnostic sensitivity to the FAT would produce less false negative results and remove the need for further testing and the associated cost. The RIAD_acetone_ test developed and validated as described herein provides one such test.

An alternative approach for resource-limited situations would be to use one of the commercial rapid immunochromatic rabies tests, commonly known as lateral flow devices, that have been developed and marketed in more recent years [10]. However, the diagnostic and analytical sensitivity of a number of rapid tests were reported as ranging from 0% to 100% for field derived samples and 32% for experimentally infected animals [10]. This high variability and the number of reported false negative results for these rapid tests, in association with the cost for countries to procure and arrange for import clearances, means that many countries, including Indonesia, have a preference to build diagnostic capacity within their laboratory network.

Within Indonesian animal health laboratories the vast majority of samples submitted for rabies diagnosis are canine brains and in DIC Bukittinggi, which is the designated National rabies reference laboratory, 87% of submitted canine brain specimens from dog bite cases submitted between 2013 and 2015 were positive. Of the 110 canine brain samples collected from apparently rabies-free areas, all samples tested negative by all tests used in our comparison. Specific immunostaining of brain smears with anti-rabies virus nucleoprotein polyclonal antisera was observed. Smears fixed with acetone or formalin were both compatible with the RIAD method; however, acetone fixation produced staining of greater clarity and intensity and produced superior DSe results. However, one advantage of formalin over acetone fixation is that it inactivates rabies virus allowing less restrictive transport and handling procedures. The staining patterns observed were identical between the RIAD_acetone_ test and FAT with both demonstrating discrete punctate staining localized to the cytoplasm of rabies infected cells. No significant non-specific or background staining was observed when antisera were used at the optimized dilutions, aiding the interpretation of results. One major advantage of the RIAD_acetone_ test over the FAT is that it may be performed in any laboratory where the SST is currently used because only light microscopy is required. It also replaces expensive antibody conjugates with readily available HRP-conjugated secondary antibodies.

Each test was comparatively evaluated using the canine brain samples from rabies endemic areas and from areas thought to be free of rabies. Whilst none of the tests returned positive results when used to test samples from presumed rabies-free regions, RIAD testing of formalin-fixed samples from endemic regions resulted in a number of false positive results when compared to the FAT and the RIAD_acetone_ test (Table 3). The RIAD_acetone_ test when compared to the SST and FAT was found to be very sensitive and of comparable accuracy to the FAT when testing dog brain samples infected with strains of rabies virus endemic to Indonesia. The FAT is designated by the OIE as the reference test for rabies because it provides “reliable results on fresh specimens…in more than 95–99% of cases” [4]. We interpreted this statement as the DSe but other interpretations are possible (e.g. DSp or predictive values). Because of the vagueness of this wording, we decided not to use priors for the DSe and DSp of the FAT to inform the Bayesian LCM. This decision was appropriate as the median posterior values for DSe and DSp for the FAT and RIAD_acetone_ were both approximately 99% and hence consistent with the reported range of values in the OIE chapter.

**Table 3:** Combinations of test results by RIAD_formalin_, RIAD_acetone_, FAT and SST from brain smears from 116 suspected rabies infected dogs in Indonesia.

RIAD Formalin	RIAD Acetone	FAT	SST	Number of Samples
+	+	+	+	56
+	+	+	−	42
+	−	−	−	5
−	+	+	+	1
−	+	+	_	1
NT	+	+	−	1
−	−	−	−	10
			Total	116

NT sample was not tested by RIAD on formalin-fixed smears

The RIAD_acetone_ test in kit form was transferred to the smaller and resource-limited Provincial laboratories for a proficiency testing round to assess its ruggedness. The test demonstrated high reproducibility with overall agreement of 97%. Of the discrepant results, one false negative was reported by one laboratory. In contrast, two false positive results that were reported by two of the participating Provincial laboratories were of less concern. Indonesian government protocols currently require samples to be sent to a National laboratory for confirmation of any negative results associated with human dog bite cases using the FAT and possibly the MIT. Adoption of the RIAD_acetone_ test instead of the SST would reduce the number of false negative samples requiring confirmatory testing. Although the evaluation of performance of the RIAD_acetone_ test demonstrated its equivalence to the FAT, it is still suggested that Provincial laboratories forward RIAD_acetone_ test-negative brain samples to the National laboratories for confirmation. This would be ongoing or until deemed unnecessary through a review of long term data generated by individual Provincial laboratories, especially those that regularly receive samples for rabies testing and hence maintain a high degree of test competency.

The RIAD_acetone_ test demonstrated diagnostic sensitivity and specificity comparable to the FAT which is the current reference (gold standard) test. It showed good reproducibility and was suitable for use in Indonesian Provincial laboratories using their existing equipment which included standard light microscopes. Replacement of the SST and/or FAT with the RIAD_acetone_ test would significantly reduce the economic burden associated with rabies virus diagnosis under the current testing regimen in Indonesia.

## Supporting information

S1 Text OpenBUGS code for estimation of the diagnostic sensitivity and specificity. (DOCX)

S1 Dataset Data used for Bayesian statistical analysis. (XLSX)
