# Supplementary material for: Development and validation of an immunoperoxidase antigen detection test for improved diagnosis of rabies in Indonesia
Source: PLoS Negl Trop Dis. 2017 Nov 13;11(11):e0006079. doi: 10.1371/journal.pntd.0006079 (PMC5703572; doi:10.1371/journal.pntd.0006079)
Supplement: S1 Text — (DOCX) [file pntd.0006079.s001.docx]

Appendix A. OpenBUGS code for estimation of the diagnostic sensitivity and specificity of RIAD acetone (test 1), FAT (test 2) and Sellers (test 3) for rabies in Indonesian dogs. Prev1 = prevalence in human bite cases and Prev2 = prevalence in survey group.

model

{

y1[1:Q, 1:Q, 1:Q] ~ dmulti(p1[1:Q, 1:Q, 1:Q],n1)

y2[1:Q, 1:Q, 1:Q] ~ dmulti(p2[1:Q, 1:Q, 1:Q],n2)

p1[1,1,1] <- Prev1*(Se1*Se2+GSe)*Se3 + (1-Prev1)*(1-Sp1)*(1-Sp2)*(1-Sp3)

p1[1,1,2] <- Prev1*(Se1*Se2+GSe)*(1-Se3) + (1-Prev1)*(1-Sp1)*(1-Sp2)*Sp3

p1[1,2,1] <- Prev1*(Se1*(1-Se2)-GSe)*Se3 + (1-Prev1)*(1-Sp1)*Sp2*(1-Sp3)

p1[1,2,2] <- Prev1*(Se1*(1-Se2)-GSe)*(1-Se3) + (1-Prev1)*(1-Sp1)*Sp2*Sp3

p1[2,1,1] <- Prev1*((1-Se1)*Se2-GSe)*Se3 + (1-Prev1)*Sp1*(1-Sp2)*(1-Sp3)

p1[2,1,2] <- Prev1*((1-Se1)*Se2-GSe)*(1-Se3) + (1-Prev1)*Sp1*(1-Sp2)*Sp3

p1[2,2,1] <- Prev1*((1-Se1)*(1-Se2)+GSe)*Se3 + (1-Prev1)*Sp1*Sp2*(1-Sp3)

p1[2,2,2] <- Prev1*((1-Se1)*(1-Se2)+GSe)*(1-Se3) + (1-Prev1)*Sp1*Sp2*Sp3

p2[1,1,1] <- Prev2*(Se1*Se2+GSe)*Se3 + (1-Prev2)*(1-Sp1)*(1-Sp2)*(1-Sp3)

p2[1,1,2] <- Prev2*(Se1*Se2+GSe)*(1-Se3) + (1-Prev2)*(1-Sp1)*(1-Sp2)*Sp3

p2[1,2,1] <- Prev2*(Se1*(1-Se2)-GSe)*Se3 + (1-Prev2)*(1-Sp1)*Sp2*(1-Sp3)

p2[1,2,2] <- Prev2*(Se1*(1-Se2)-GSe)*(1-Se3) + (1-Prev2)*(1-Sp1)*Sp2*Sp3

p2[2,1,1] <- Prev2*((1-Se1)*Se2-GSe)*Se3 + (1-Prev2)*Sp1*(1-Sp2)*(1-Sp3)

p2[2,1,2] <- Prev2*((1-Se1)*Se2-GSe)*(1-Se3) + (1-Prev2)*Sp1*(1-Sp2)*Sp3

p2[2,2,1] <- Prev2*((1-Se1)*(1-Se2)+GSe)*Se3 + (1-Prev2)*Sp1*Sp2*(1-Sp3)

p2[2,2,2] <- Prev2*((1-Se1)*(1-Se2)+GSe)*(1-Se3) + (1-Prev2)*Sp1*Sp2*Sp3

LowerGSe <- (Se1 - 1)*(1 - Se2)

UpperGSe <- min(Se1,Se2) - Se1*Se2

Se1~dbeta(1,1)

Se2~dbeta(1,1)

Se3~dbeta(1,1)

Sp1~dbeta(1,1)

Sp2~dbeta(1,1)

Sp3~dbeta(1,1)

Prev1~dbeta(1,1)

Z ~ dbern(tau0)

Pistar ~ dbeta(1.27, 274.82) ## Mode=0.001, 90% sure pistar < 0.01

Prev2 <- Z * Pistar

Se12diff<-Se1-Se2

PrSe12<-step(Se12diff)

Se13diff<-Se1-Se3

PrSe13<-step(Se13diff)

Se23diff<-Se2-Se3

PrSe23<-step(Se23diff)

GSe ~ dunif(LowerGSe, UpperGSe)

}

# data

list(n1=116,n2=80,Q=2,tau0=0.9, y1=structure(.Data=c(57,44,0,0,0,0,0,15),.Dim=c(2,2,2)), y2=structure(.Data=c(0,0,0,0,0,0,0,80),.Dim=c(2,2,2)))

# initial values

list(Se1=0.9, Sp1=0.99, Se2=0.9, Sp2=0.99, Se3=0.9, Sp3=0.99, Prev1=0.5, Z=0, GSe=0.01)

list(Se1=0.95, Sp1=0.95, Se2=0.95, Sp2=0.95, Se3=0.8, Sp3=0.95, Prev1=0.8, Z=1, GSe=0.001)
